# Nutritional Content and Antioxidant Capacity of the Seed and the Epicarp in Different Ecotypes of *Pistacia atlantica* Desf. Subsp. *atlantica*

**DOI:** 10.3390/plants9091065

**Published:** 2020-08-19

**Authors:** Amina Labdelli, Abdelkrim Rebiai, Mohammed Tahirine, Ahmed Adda, Othmane Merah

**Affiliations:** 1 Scientific and Technical Research Centre for Arid Areas (CRSTRA), BP 1682 RP, 07000 Biskra, Algeria; aminalabdelli@yahoo.fr (A.L.); tarektahirine@gmail.com (M.T.); 2 Laboratory of Agro-Biotechnology and Nutrition in Semi-Arid Areas, Ibn Khaldon University, 14000 Tiaret, Algeria; adda2ahmed@yahoo.fr; 3 VTRS Laboratory, University of El-Oued, P.O. Box 789, 39000 El-Oued, Algeria; new.rebiai@gmail.com; 4 Laboratoire de Chimie Agro-industrielle (LCA), Université de Toulouse, INRA, INPT, 31030 Toulouse, France; 5 Département Génie Biologique, Université Paul Sabatier, IUT A, 32000 Auch, France

**Keywords:** phenolic composition, antioxidant activity, Atlas pistachio, ecotypes diversity, seed, epicarp

## Abstract

Phenolic compounds are secondary metabolites that occur naturally in all plants. Seeds are among the richest organs of plants in phytochemicals, vitamins and minerals. These compounds and their biological activities are of great importance for human health. This study aimed to analyze the phenolic composition and their antioxidant activity in the seeds and epicarps of six Algerian populations of *Pistacia atlantica* Desf. subsp. *atlantica* growing along an aridity gradient from semi-arid to Saharan environmental conditions. Higher phenolic contents were observed in epicarp compared to seeds whatever the ecotype. The highest phenolic content of seeds and epicarps was observed in ecotype of Djelfa and the lowest values in Tiaret (T-Z). Phenolic composition, measured by reversed-phase high-performance liquid chromatography (HPLC), showed that quercetin in epicarp, gallic and chlorogenic acids in seeds were the most present in all ecotypes. Large differences were observed between ecotypes for nutritional values. Seeds were rich in flavonoids, proteins, carbohydrates and essential elements such as potassium, calcium, phosphorus and iron. These results highlighted the potential importance of Atlas pistachio fruits as a source of essential compounds that contribute to human health. Moreover, this underused species may serve a potential source for antioxidant components for alimentation and cosmetics purposes.

## 1. Introduction

Atlas pistachio (*Pistacia atlantica* Desf.) is an Anacardiaceae that extends from southwestern Asia to northwestern Africa [1]. In Algeria, this species is less known than *Pistacia vera* L., and grows in the wild in the sub-humid zones as well as in the Saharan regions [2]. Its oil is used for both food and cosmetic purposes [3,4]. Nevertheless, fruits of Atlas pistachio are widely consumed by the local population as a nutrient, and they have numerous applications in cosmetics, pharmaceutical and feed industry [5,6].

Properties such as protection against coronary heart disease, anti-inflammatory activity, and anti-tumor are often associated with phenolic compounds. The different parts of this tree have traditionally been used for various purposes, providing seeds rich in nutrients and natural bioactive compounds, in addition to a high content of vitamins and minerals [3,6,7,8]. The medicinal properties of this species are known for potent pharmacological activities, low toxicity and economic viability [9]. The potential of the antioxidant constituents of plant materials for the maintenance of health and protection against coronary heart diseases and cancer also arouses interests [10,11].

Synthetic antioxidants have aspect effects; previous research indicates the relation between the long-term intake of synthetic antioxidants and some health problems, like gastrointestinal tract problems, skin allergies and in some cases augmented the risk of cancer [12,13]. These studies reported that high doses of synthetic antioxidants like butylhydroxyanisole (BHA) and butylhydroxytoluene (BHT) are tumor promoters. Unlike BHA and BHT, α-Tocopherol (vitamin E) is non-carcinogenic [14]. Therefore, it seems important to set the objective of replacing synthetic antioxidants with natural antioxidants for food production uses, cosmetics, pharmaceutical and agricultural purposes [15,16]. The use of natural antioxidants allows producers to satisfy the demands of consumers for cleaner-label products with exclusively natural constituents. Large amounts of data have been generated on the antioxidant characteristics of food plants worldwide [17,18].

Seeds are excellent sources of many essential nutrients, including minerals, vitamins, antioxidants, and phytochemicals [19]. Minerals, either macro- or micronutrients, in sufficient quantities are necessary for the proper functioning of the human body [20]. Damage caused by the mineral deficiency may be associated with stunted growth and cognitive impairment in the population [21,22].

Some reports have studied the biochemical composition, mostly the phenolic content and its antioxidant activity of Atlas pistachio on fruits [6,19,23]. Other studies involved *P. atlantica* subsp. *kurdica* or subsp. *mutica* [24,25]. To our knowledge, there are no detailed studies on the antioxidant activity and phenolic profile of the epicarp and seed of *P. atlantica* Desf. subsp. *atlantica* growing in Algeria. These seeds have not been fully characterized. The objective of this study was to evaluate the protein, sugar contents and mineral composition of seeds of several ecotypes from six different regions in Algeria representing gradient aridity from semi-arid to Saharian. The polyphenol content, phenolic profile by reversed-phase high-performance liquid chromatography (HPLC) and antioxidant capacity of seeds and epicarps were also studied.

## 2. Results

### 2.1. Biochemical Analysis

#### 2.1.1. Protein and Soluble Sugar Content of Seeds

Table 1 shows the protein and soluble sugar contents of fruits. These traits were strongly influenced by the used ecotypes (*p* ≤ 0.05). Among all the samples analyzed, the ecotype of Batna showed the highest content of sugar while Bechar indicated the lowest content (Table 1). However, Tiaret (T-R) and Batna ecotypes indicated the highest and lowest protein content in fruits, respectively (Table 1).

#### 2.1.2. Mineral Analysis

Table 2 presents the ash and mineral element contents of seeds from six ecotypes from different regions in Algeria. Minerals varied according to the origin of the Atlas pistachio fruits (Table 2). High potassium and calcium contents were observed whatever the ecotype. Potassium was found in large quantities in Laghouat fruits, and low content was reported in the fruits of Djelfa. The fruits of Batna were very rich in Ca, and those of Laghouat were the poorest compared to the other populations (Table 2). Iron was in significant concentration in the fruits of these ecotypes (Table 2).

#### 2.1.3. Total Phenolic Content

**Figure 1:**
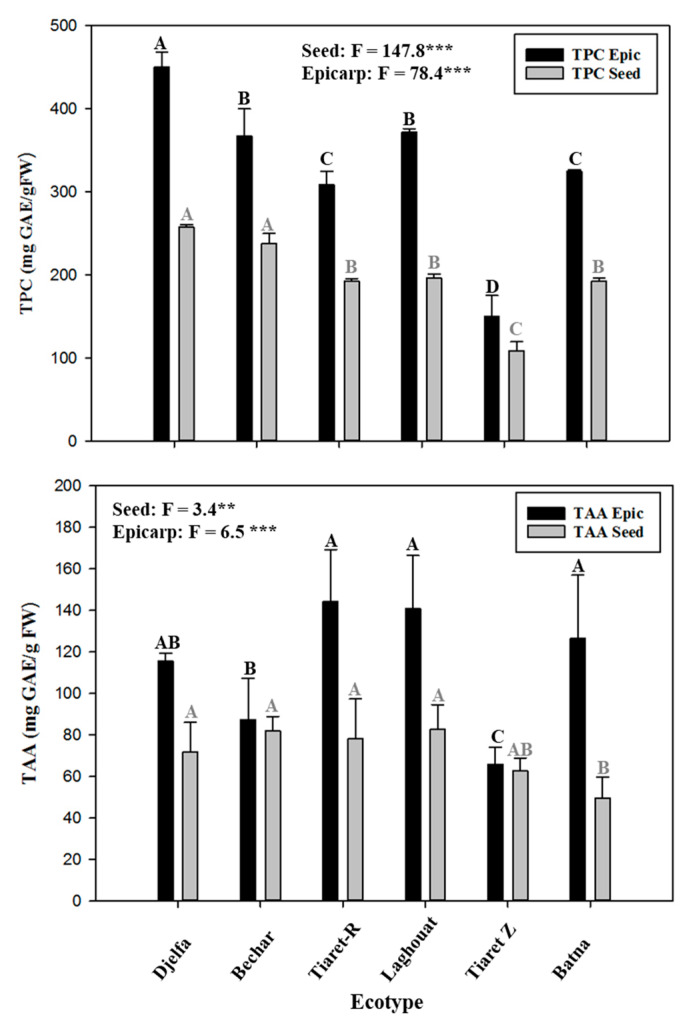
Mean values of total phenolic contents (TPC) and total antioxidant activity (TAA) in epicarp and seed of Atlas pistachio harvest in six regions in Algeria. Bars represent standard deviations. F values are displayed for each trait and each part of the fruit. Columns with the same letter are not significantly different at *p* < 0.05 probability level according Duncan test. Dark letters concern the comparison of TPC and TAA of epicarp between six ecotypes of *P. atlantica* Desf. Gray letters indicate significance for TPC and TAA of seed between six ecotypes of *P. atlantica* Desf. ** and *** significant at 0.01 and 0.001 probability, respectively.

A significant difference was observed between ecotypes for the total polyphenolic compounds and total antioxidant activity of seed and epicarp (Figure 1). The epicarp content of phenolic compounds was significantly higher than seed whatever the ecotype. It was observed that epicarp was a rich natural source of polyphenols. The highest phenolic concentration in epicarp and seed was observed in fruits of Djelfa and the lowest in Tiaret (T-Z) (Figure 1).

#### 2.1.4. Phenolic Compound Identification

Some selected phenolics were separated and identified by comparison with authentic standards using reversed-phase high-performance liquid chromatography (HPLC) (Figure S1, Table S1). The quantitative and qualitative analyses of phenolic extracts were performed by HPLC (Figure S2, Table 3). The results indicated that the quercetin was the main flavonoid constituent identified in both seed and epicarp. Gallic acid, chlorogenic acid and rutin were present in all studied ecotypes. The highest flavonoids content was observed in the epicarp of Djelfa, and the lowest value was measured in the seeds of Tiaret (T-Z) (Table 3). Epicarp of Djelfa showed the highest content of total phenolis acids compared with those of other ecotypes, while Batna ecotype showed the lowest value. The vanillin content was very low, only the Djelfa seeds recorded the highest value.

### 2.2. Total Antioxidant Activity (TAA)

The highest value of total antioxidant activity (TAA) was observed in epicarp of Tiaret (T-R) and the lowest ones on the seed of Batna (Figure 1). Moreover, TAA of epicarps was significantly higher than those of seeds. This activity was almost double for all ecotypes, except in Bechar and Tiaret (T-Z) that it was low and identical (Figure 1).

## 3. Discussion

The protein content (Table 1) showed similar levels to those previously reported in literature [26,27] and estimated between 8–10%. The proteins synthesized and stored in the mature seed are broken down into free amino acids to produce the energy needed for seed growth but also germination [28].

The results of the reducing sugars showed that these seeds have accumulated high levels of these reserves (Table 1). The importance of sugar level in the fresh vegetable soybean seeds because the total soluble sugar content directly influences the organoleptic properties of edamame and determines consumer acceptability, and there is a significant relationship between taste score and sugar content [29,30,31,32].

Moisture is an important factor associated with climate and soil. In the results (Table 2), it is clear from sample A that moisture was low compared to the rest of the ecotypes. This could be explained by the fact that region Ais characterized by higher temperature and less rainfall than the other studied regions.

The ash content of seed varied nearly two times between extreme ecotypes (Table 2). These values were highest than those reported by Mohammadi et al. [19] and similar to those already reported by Saffarzadeh et al. [26].

Potassium and calcium were the most abundant macronutrients in the fruits of *P. atlantica* Desf., [19,26], this was confirmed in our current study. The results obtained indicated that these fruits contain high levels of potassium and reached 15.83 ± 0.25 g/kg, which is significantly higher than that obtained for (7 g/kg) in *P. atlantica* Desf. and (8.8 g/kg) in *Pistacia khinjuk* [19,26].

The values obtained from calcium and phosphorus content were nearly three times higher than those mentioned in previous studies. Indeed, the amount of calcium 3.76 ± 0.10 g/kg was higher than those (1.13 to 1.36 g/kg) reported in previous works [19,26].

The results of sodium and magnesium contents were quite similar to values previously reported in literature and ranged from 0.34 to 0.51 g/kg [19,26].

Fruits of Algerian Atlas pistachio contained high levels of selenium and iron. Similar results were observed in *P. terebinthus* L. [33]. The maximum contents of selenium 0.82 g/kg and iron 0.056 g/kg were witnessed in samples B and T-R, respectively. Our results of the micronutrients namely Mn, Zn, Pb, and Cu agree with those previously reported [19,33].

The differences in the concentrations of mineral element in seeds between all ecotypes (Figure 1) could be explained by soil variability, growing conditions, genetic factors [34,35,36]. The minerals are used in tissue structure and play a role in cellular metabolism and their pro-oxidant and health benefits [37,38].

The six regions were characterized by different climatic and edaphic conditions [2,39,40] (Table S2). The station of Bechar was characterized by low rainfall (111 mm) and high temperatures compared to the other stations. At the opposite, Banta recorded the highest rainfall (210 mm) and the lowest temperatures.

Mineral contents in ecotypes A and B were approaching 5% (Table 2). Both were from the hyper-arid and semi-arid climatic regions. As for the rest of the ecotypes, minerals content values were lower and convergent. They were from arid, semi-arid, and fresh arid climatic regions. The climate can affect the content of minerals [34,35,36]. The more arid climate, the more minerals are contained in seeds. Soil quality can also be an influencing factor as the seeds of plants in clay soils are richer in minerals.

The existence of differences in the concentrations of mineral elements in seeds at different geographic zones can be explained by soil and climatic conditions variability (Table S2). According to Penfield and MacGregor [41] and Bouabdelli et al. [42], nutrient content is strongly influenced by the geographical origin of the seeds, environmental conditions of the mother plant during seed development and genetic factors.

Through the results obtained in this study, which indicate the richness of *P. atlantica* Desf. in the minerals, this species can be part of the diet and provide the human body with its needs of minerals. The human body needs daily 1000 mg of Calcium, 0.9 mg Copper, 8 mg Iron, 400 mg Magnesium, 2.3 mg Manganese, 700 mg Phosphorus, 55 µg Selenium, 11 mg Zinc, 3400 mg Potassium, and 1500 mg Sodium [43]. Optimal intakes of these minerals reduce individual risk factors, such as those associated with cardiovascular disease [44]. Trace elements, although needed in very small amounts, also play important functions. They are key protein components such as hemoprotein and hemoglobin that play a crucial role in biochemical functions and essential enzyme systems [45]. Fruits rich in phosphorus and calcium are essential for bone and tooth development [46].

The total phenolic content of seed and epicarp in each ecotype of Atlas pistachio is shown in Figure 1. The phenolic content of epicarp was significantly higher than those of seed (nearly twice, Figure 1). Fruits of the Atlas pistachio are rich in lipids, particularly unsaturated fatty acids (UFA) [4,5] and have significant antioxidant capacity [6,47]. Indeed, the biochemical analysis of the fruits of the different ecotypes indicates that they were rich in phenolic compounds (Figure 1) at the scale of the seed and epicarp [24,47]. However, the contents of these compounds remain very variable according to the different origins of the studied ecotypes. The results emphasized high antioxidant activity for all ecotypes (Figure 1). Similar results were observed by previous studies [23,25], and estimated this amount in the range of 149–286 mg gallic acid equivalents (GAE)/g dry matter (DM). Other studies investigated TPC in Pistachio (*Pistacia vera* L.) hull (21.3–39.3 mg/g gallic acid equivalents (GAE)) and the leaves of *P. atlantica* Desf. (71.86–514.81 mg gallic acid equivalents (GAE)/g dried extract) [48,49]. However, these data are not comparable to our results because they concern another species [48] or another organ such as the leaves [49]. Mohammadi et al. [19] have shown that hull of *P. atlantica* Desf. is rich in vitamin C as a natural antioxidant. This richness in antioxidants results in the maintain of seed dormancy by limiting water and oxygen diffusion to almond [50].

Total phenols consisted of phenolic acids, vanillin and flavonoids, and their level depends on growth stage and on species (or subspecies) [47,48,49,50,51,52]. These compounds can delay or inhibit the oxidation of lipids by impeding the initiation or propagation of oxidative chain reactions [53]. They are commonly used in food industry as potential inhibitors of lipid peroxidation [8]. The antioxidant activity of phenolic compounds is mainly due to their redox properties, which can play an important role in the absorption and neutralization of free radicals, the extinction of singlet, triplet oxygen, or peroxides in decomposition [54,55]. These natural compounds can reduce oxygen concentration and therefore exerting their beneficial effects on health [56,57].

The Atlas pistachio fruits were very rich in flavonoids especially their epicarp (Table 3). Epidemiological studies suggest that the consumption of flavonoid-rich foods protects against human diseases which are associated with oxidative stress [58]. Indeed, it is well known that the consumption of fruit rich in phytochemicals such as polyphenols, carotenoids and vitamins E and C reduce the risk of cancer and cardiovascular diseases [54,59,60].

A positive correlation was observed between total antioxidant activity and total phenolic content in two parts of the *P*. *atlantica* Desf. seed. The results also showed that the antioxidant activity determined at the seed level correlated positively and significantly with its polyphenols content (r = 0.73, *p* ≤ 0.05) (Figure S3). This result mirrored that the majority (53%) of antioxidant capacity results from the contribution of phenolic and flavonoid compounds. Thus, the antioxidant activity of the extracts is not limited to phenolic compounds. The activity may also imply the presence of other secondary antioxidant metabolites, such as volatile compounds, carotenoids, coumarins and vitamins. The antioxidant activity of phenols is mainly due to their redox properties, which enable them to act as reducing agents, hydrogen donors and singlet oxygen extinguishers. They may also have the potential for metal chelation [55].

Concerning the biochemistry of *P. atlantica* Desf., no data have been reported previously on the qualitative and quantitative composition of seeds and epicarps of this species under different climatic and edaphic conditions in Algeria. This study highlighted the intra-population variability for biochemical traits in this species. These differences may also result from interaction between environmental and genetic factors.

## 4. Materials and Methods

### 4.1. Geographical Origin of Seeds

**Figure 2:**
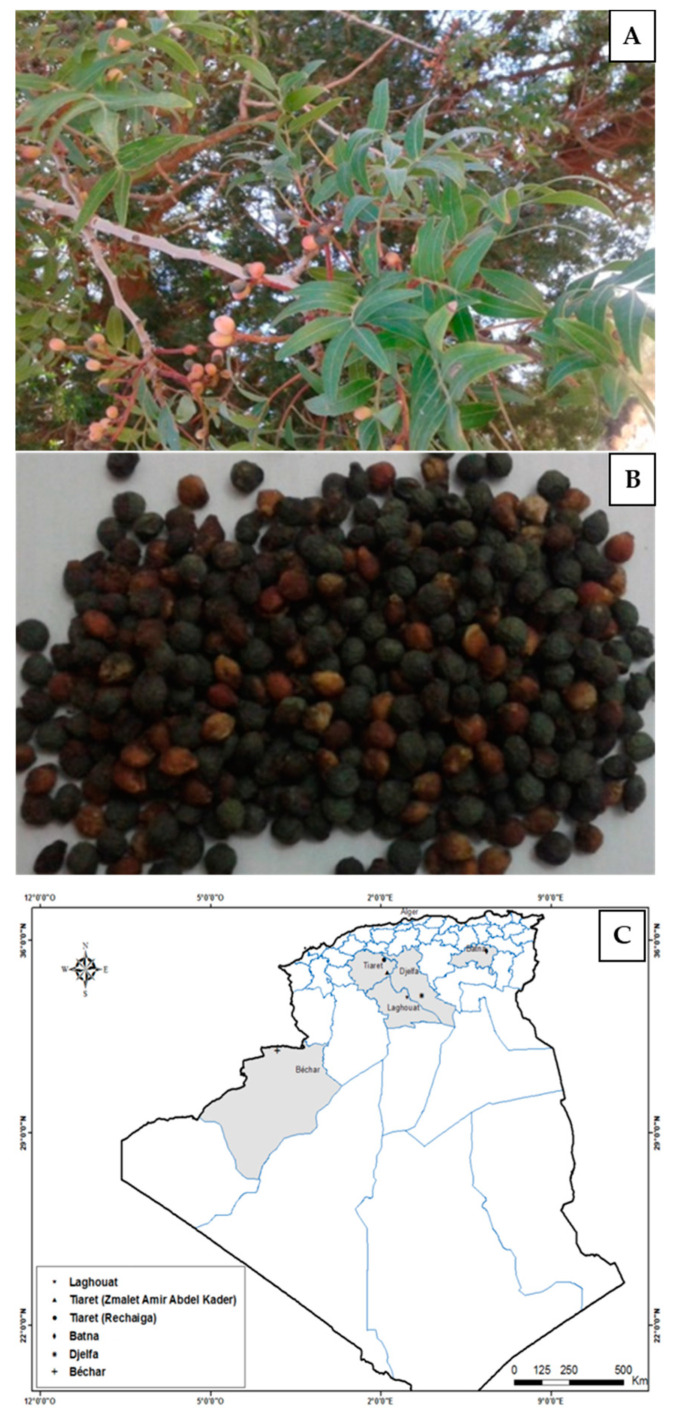
Pictures of tree (**A**) and fruits (**B**) of *P. atlantica* Desf. and localization (**C**) of the different studied ecotypes in Algeria (**B**).

Healthy seeds of *P. atlantica* Desf. ssp. *atlantica* were collected on three hectares, at full ripening in six different locations in Algeria at the beginning of October to November 2016 (A: Bechar, B: Batna, D: Djelfa, L: Laghouat; T-R: Tiaret Rechaiga, T-Z: Tiaret Zemalet El Emir Abdelkader) (Figure 2). Epicarp was manually separated from seeds.

The fruits were collected randomly, according to the method of the transect, from thirty trees and were stored in the laboratory at 4 °C till extraction. Briefly, on the experimental area, we took three lines according to the method of Waddell; every line is named a transect [4,61].

### 4.2. Chemicals and Reagents

All reagents were of analytical grade. 3,4,5-trihydroxybenzoic acid (Gallic acid; GA), 3,4-dihydroxy-trans-cinnamate (Caffeic acid; CA), 3,5,7,3′,4′-pentahydroxyflavone (Quercetin; QC), 3-(3,4-dihydroxycinnamoyl) quinic acid (Chlorogenic acid; CGA), trans-4-Hydroxycinnamic acid (*p*-Coumaric acid; *p*-C), 4-hydroxy-3-methoxybenzoic acid (Vanillic acid; VA), quercetin-3-O-rutinoside (Rutin; RU), 4-hydroxy-3-methoxybenzaldehyde (Vanillin; V), anthrone, glucose, and hydrochloric acid (HCl) were procured from Sigma-Aldrich (St. Louis, MO, USA). Sodium phosphate (Na_3_PO_4_, anhydrous, powder, extra pure), sodium hydroxide (NaOH), sulfuric acid (H_2_SO_4_), hydrogen peroxide (H_2_O_2_), potassium sulfate (K_2_SO_4_), ammonium sulfate (NH_4_)_2_SO_4_, aluminum chloride (AlCl_3_) and sodium carbonate (Na_2_CO_3_) were purchased from Prolabo (Paris, France). Folin-Ciocalteu reagent (FCR), acetone (Me_2_CO), chloroform (CHCl_3_) and methanol (MeOH) were obtained from BIOCHEM chemopharma Co (France). High purity water was used in all experiments. Acetonitrile of HPLC-gradient grade was purchased from Sigma-Aldrich (St. Louis, MO, USA).

### 4.3. Biochemical Analysis

#### 4.3.1. Nitrogen, Protein Contents and Soluble Sugars Content

Nitrogen content was analyzed by Kjeldahl method [62]. Protein content was determined as nitrogen content multiplied by 6.25.

The soluble sugars content was quantified by anthrone method [63]. Soluble sugars content was expressed as mg glucose equivalent per gram of fresh weight (mg/gFW).

#### 4.3.2. Mineral Analysis

Moisture content was determined by over drying the sample (5 to 9 g) at 103 ± 2 °C until reaching a constant weight. Then, the samples were placed in a desiccator and cooled to room temperature. The moisture content was expressed as a percentage of the fresh weight of the samples [64].

Twelve macro and micro elements were determined in the grains from six different stations. One gram of ground seeds was incinerated in a muffle furnace at 500 °C. The ashes obtained after they were mineralized and dissolved in a sand bath in pure hydrochloric acid (HCl) for 15 min then filtered through filter paper to complete the volume to 100 mL with distilled water [65]. The levels of copper (Cu), manganese (Mn), zinc (Zn), selenium (Se), lead (Pb) and iron (Fe) were determined by atomic absorption spectrometer (Perkin Elmer, PinAAcle 900T, Waltham, MA, USA), sodium (Na) and potassium (K) by flame spectrophotometry (Jenway, Models PFP7, Essex, UK), calcium (Ca), and magnesium (Mg) by titration. Phosphorus (P) concentration of seed was assessed by spectrophotometric determination at 650 nm against a standard range [66].

Nitrogen (N) was determined by the Kjeldahl method (Kjeltec™ 8400 and Passeur Kjeltec™ 8420, Nanterre, France) by mineralization of organic nitrogen into mineral nitrogen [67].

#### 4.3.3. Total Phenolic Content

##### Plant Sampling and Preparation for Extract

Methanol was found as the most efficient solvent for phenolic extraction according to previous works [6,23,50]. One gram of seed and epicarp were crushed separately into powder using a mortar grinder (RM 200-Retsch, Haan, Germany) with 20 mL of methanol and stirred for 48 h in dark at room temperature. The mixture was filtered through a hydrophilic polypropylene (GHP) filter with 0.45 µm pores; the solvent was eliminated under reduced pressure in a rotary evaporator at 60 °C. The residue (crude extract) was dissolved in 3 mL of methanol for analysis [23,25,50].

Total phenolic content was determined using Folin-Ciocalteu reagent (FCR) according to the method of Singleton et al. [68]. Results were expressed as milligram of gallic acid equivalent per gram of fresh weight (mg GAE/gFW).

#### 4.3.4. Chromatographic Separation of Phenolic Compounds by HPLC

The chromatographic system for separation, analysis of phenolic acids and flavonoids were carried out with Shimadzu model Prominence liquid chromatography, thermostatic column compartment, online degasser and a UV-visible detector model SPD-20A. An analytical column used was a Shim-pack VP-ODS C18 (4.6 mm × 250 mm, 5 µm), (Shimadzu Co., Kyoto, Japan). The volume injected was 20 µL.

### 4.4. Total Antioxidant Activity (TAA)

The total antioxidant activity of the extract was evaluated by the phosphomolybdenum method according to the procedure described by Prieto et al. [69]. The total antioxidant activity was expressed as mg gallic acid equivalent per gram of fresh weight (mg GAE/gFW).

### 4.5. Statistical Analyses

Results were given as mean values ± standard deviations (SD) of 3 replicates. The data were not log-transformed. The comparison of quantitative variables was performed using one-way analysis of variance (ANOVA), by STATISTICA software package (StatSof, Tulsa, OK, USA). Mean comparison was performed using Duncan test at 5% probability level of significance.

## 5. Conclusions

The phenolic profile and antioxidant activity in the seeds and epicarp of *P. atlantica* Desf. subsp. *atlantica* showed that these six populations differed significantly. The seed and epicarp of the Djelfa station exhibited the highest phenolic concentration. at the opposite, the seed and epicarp of the Tiaret station (T-Z) presented low levels of polyphenols. In general, the seeds of Atlas pistachio used in traditional medicine are very rich in flavonoids, carbohydrates and essential mineral elements and can be used as nutritional foods. This species therefore serves as a natural local source of dietary antioxidants beneficial to human health. Further investigations are needed, particularly concerning the effect of phenols on human health to ascertain their impact on local populations in Algeria.

## Figures and Tables

**Table 1:** Mean values of Protein and soluble sugar contents in seeds of Atlas pistachio harvested in six regions in Algeria. Test F values are displayed and the significance of origin seeds.

Parameters	A	B	D	L	T-R	T-Z	Test F
Proteins (%)	7.73 ± 0.13 ^b^	7.69 ± 0.55 ^b^	8.45 ± 0.20 ^a^	8.82 ± 0.35 ^a^	9.56 ± 0.32	8.46 ± 0.42 ^a^	11.57 ***
Soluble sugars (mg/gFW)	128.06 ± 8.39 ^b^	55.24 ± 5.02 ^c^	125.29 ± 9.55 ^b^	94.96 ± 4.59 ^a^	77.17 ± 7.85 ^d^	93.16 ± 5.57 ^a^	46.54 ***

A: Bechar, B: Batna, D: Djelfa, L: Laghouat; T-R: Tiaret-Rechaiga, T-Z: Tiaret-Zemalet El Emir Abdelkader; Within each line, lowercase letters indicate significance at *p* < 0.05 probability level according Duncan test. *** significant at 0.001 probability.

**Table 2:** Mineral composition (mg/g) of seeds of six ecotypes of *P. atlantica* Desf. harvested in different geographic areas in Algeria. Test F values are displayed and the significance of origin seeds.

Parameters	A	B	D	L	T-R	T-Z	F Value
Moisture (%)	3.2	4.03	3.72	4.38	4.5	4.1	-
Ash (%)	5.03 ± 0.84 ^b^	5.12 ± 1.10 ^b^	3.29 ± 0.01 ^a^	3.66 ± 0.74 ^a^	3.99 ± 0.38 ^a^	3.42 ± 0.10 ^a^	2.85 ns
N (%)	1.24 ± 0.02 ^b^	1.23 ± 0.09 ^b^	1.35 ± 0.03 ^a^	1.41 ± 0.06 ^a^	1.53 ± 0.05 ^c^	1.35 ± 0.07 ^a^	11.57 ***
P	2.42 ± 0.03 ^e^	1.60 ± 0.02 ^a^	2.00 ± 0.003 ^c^	2.32 ± 0.01 ^d^	1.80 ± 0.02 ^b^	2.44 ± 0.01 ^f^	3235.1 ***
Ca	2.57 ± 0.06 ^d^	3.77 ± 0.23 ^a^	2.35 ± 0.06 ^c^	1.40 ± 0.00 ^b^	3.76 ± 0.10 ^a^	2.99 ± 0.10 ^e^	191.86 ***
Na	0.29 ± 0.05 ^c,d^	0.23 ± 0.05 ^b,c^	0.34 ± 0.05 ^d^	0.19 ± 0.05 ^a,b^	0.11 ± 0.0 ^a^	0.19 ± 0.05 ^a,b^	8.87 **
K	12.68 ± 0.25 ^b^	12.11 ± 0.24 ^a^	15.83 ± 0.25 ^e^	10.18 ± 0.25 ^c^	12.29 ± 0.25 ^a,b^	14.06 ± 0.14 ^d^	200.09 ***
Mg	0.37 ± 0.01 ^d^	0.24 ± 0.01	0.18 ± 0.01 ^a^	0.13 ± 0.01 ^b^	0.18 ± 0.06 ^a^	0.19 ± 0.01 ^a^	34.48 ***
Se	0.43	0.82	0.22	0.15	0.47	0.17	-
Fe	0.051	0.01	0.05	0.019	0.056	0.026	-
Mn	0.005	0.002	0.008	0.001	0.002	0.002	-
Zn	0.002	0.003	0.002	0.001	0.001	0.001	-
Cu	0.005	0.003	0.004	0.005	0.005	0.005	-
Pb	0.002	0.006	0.012	0.014	0.001	0.017	-

A: Bechar, B: Batna, D: Djelfa, L: Laghouat; T-R: Tiaret-Rechaiga, T-Z: Tiaret-Zemalet El Emir Abdelkader; Within each line, lowercase letters indicate significance at *p* < 0.05 probability level according Duncan test. ** and *** significant at 0.01 and 0.001 probability, respectively.

**Table 3:** Phenolic compounds content (mg/gFW) identified by reversed-phase high-performance liquid chromatography (HPLC) in epicarp and seed in different ecotypes of *P. atlantica* Desf. harvested in Algeria. ND: not determined.

Polyphenols (mg/gFW)	Ecotype	Phenolic Acids						Flavonoids				Phenolic Aldehyde
		Caffeic Acid	Chlorogenic Acid	*p*-Coumaric Acid	Gallic Acid	Vanillic Acid	Total Phenolic Acids	Quercetin	Rutin	Naringin	Total Flavonoids	Vanillin
Epicarp	A	ND	0.92	ND	1.62	ND	2.54	23.39	0.21	ND	23.6	ND
	B	0.02	0.13	ND	0.04	0.02	0.21	104.13	0.25	0.09	104.48	0.09
	D	0.39	43.86	0.28	48.8	0.82	94.15	452.89	5.46	1.51	459.86	0.07
	L	0.15	16.28	0.11	9.65	0.06	26.26	158.57	1.16	0.23	159.96	0.03
	T-R	ND	2.79	0.02	3.26	ND	6.07	46.76	0.19	0.22	47.17	0.02
	T-Z	ND	10.79	0.03	5.91	0.05	16.77	25.74	1.48	0.07	27.29	0.04
Seed	A	0.03	9.7	0.15	6.44	0.07	16.39	41.8	1.13	0.3	43.23	ND
	B	0.01	3.48	0.03	1.46	ND	4.99	42.84	0.13	0.21	43.18	ND
	D	ND	9.83	0.16	6.28	0.09	16.36	43.99	1.02	0.07	45.08	0.11
	L	ND	8.01	0.15	4.51	0.03	12.7	32.26	0.72	0.16	33.15	0.02
	T-R	0.02	6.09	0.07	2.51	ND	8.69	39.46	0.4	0.13	39.98	ND
	T-Z	ND	4.31	0.04	1.27	0.03	5.64	13.35	0.39	0.08	13.82	ND

A: Bechar, B: Batna, D: Djelfa, L: Laghouat; T-R: Tiaret-Rechaiga, T-Z: Tiaret-Zemalet El Emir Abdelkader.

## Supplementary Materials

The following are available online at https://www.mdpi.com/2223-7747/9/9/1065/s1, Figure S1: High-performance liquid chromatography (HPLC) chromatogram of phenolic compounds. Identified compounds are: GA—gallic acid; CGA—Chlorogenic acid; VA—Vanillic acid; CA—Caffeic acid; V—Vanillin; *p*-CA—*p*-coumaric acid; RU—Rutin; QC—quercetin. Figure S2: Phenolic compounds content identified by High-performance liquid chromatography (HPLC) chromatogram in epicarp of Djelfa (D). Identified compounds are: GA—gallic acid. CGA —Chlorogenic acid. VA—Vanillic acid. CA—Caffeic acid. V—Vanillin. *p*-CA—*p*-coumaric acid. RU—Rutin. NAR—naringin and QC—quercetin. Figure S3: Correlation between antioxidant activity (TAA) and polyphenol content (TPC) in tow part of fruits of *Pistacia atlantica* Desf. harvested in six geographic areas in Algeria. Table S1: Retention times (Rt), calibration curves, regression coefficients, Detection and quantification limits for phenolic compounds. Table S2: Ecological factors of the *Pistacia atlantica* Desf. collection sites.
