# Supplementary material for: Nutritional Content and Antioxidant Capacity of the Seed and the Epicarp in Different Ecotypes of Pistacia atlantica Desf. Subsp. atlantica
Source: Plants (Basel). 2020 Aug 19;9(9):1065. doi: 10.3390/plants9091065 (PMC7570190; doi:10.3390/plants9091065)
Supplement: Supplementary file 1 [file plants-09-01065-s001.pdf]

S1. Chromatographic Separation of Phenolic Compounds by HPLC

Samples preparation

The extracts of epicarp and seed (10 mg) were dissolved in HPLC grade methanol (10 mL). The sample solutions were filtered with 0.45 μm Millipore nylon filter disk. Then 20 μL of the sample was analyzed in the HPLC system.

Standard Solutions

A stock solution of polyphenol was prepared by dissolving 10 mg of purified polyphenol in a 50 mL volumetric bottle containing a sufficient volume of methanol (HPLC grade) to dissolve the polyphenol, it was sonicated for about 10 min and then brought to volume with mobile phase. Daily working standard solutions of polyphenols were prepared by proper dilution of the one with the mobile phase. Any of these solutions (20 μL) was injected three times into the column, the peak area and retention times were recorded.

Chromatographic Conditions

The chromatographic system for the separation, analysis of phenolic acids and flavonoids were carried out with shimadzu model prominence liquid chromatography, thermostatic column compartment, online degasser and an UVvisible detector model SPD-20A (operating at 268 nm). An analytical column used was a Shim-pack VP-ODS C18 (4.6 mm × 250 mm, 5 μm), (Shimadzu Co., Japan). A binary gradient linear system consisting of acetonitrile — A and 0.2% acetic acid in water — B was used. Gradient method was generated by starting with 90% B; then decreasing to 86% B in 6 min, to 83% B in 16 min, to 81% B in 23 min, to 77% B in 28 min, held at 77% B in 28–35 min, to 60% B in 38 min, to 90% B in 50 min; at a flow rate of 1 mL/min. Quantification of separated peaks was performed by calibration with standard gallic acid (GA), chlorogenic acid (CGA), vanillic acid (VA), caffeic acid (CA), *p*-coumaric acid (*p*-CA), vanillin (V), rutin (RU), naringin (NAR), and quercetin (QC). The phenolic composition was quantified by plotting a standard curve with the respective standards.

LOD and LOQ

Detection Limit is the lowest concentration in a sample that can be measured but not generally quantified under the experimental conditions specified. The quantitation limit is the lowest analyte concentration in a sample, which can be determined with acceptable accuracy and precision. The values for LOD and LOQ are given in Table S1.

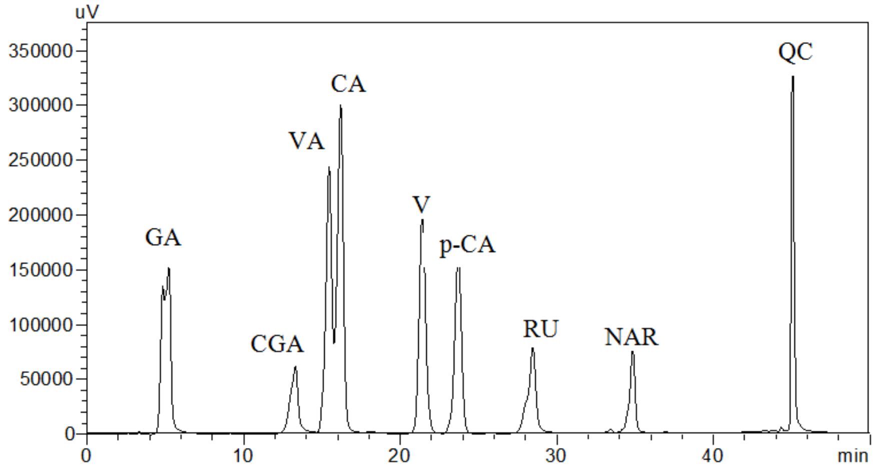

Figure S1. High-performance liquid chromatography (HPLC) chromatogram of phenolic compounds. Identified compounds are: GA—gallic acid. CGA —Chlorogenic acid. VA—Vanillic acid. CA—Caffeic acid. V—Vanillin. *p*-CA—p-coumaric acid. RU—Rutin. NAR—naringin and QC—quercetin.

Table S1. Retention times (Rt), calibration curves, regression coefficients, detection and quantification limits for phenolic compounds.

N°	Compound	Rt (min)	Calibration curve	R^2	LOQ (µg/mL)	LOD (µg/mL)	Range(µg/mL)
01	Gallic acid	5.29	Y = 23616X − 7232	0.9986	0.36939	0.12189	2.5 – 300
02	Chlorogenic acid	13.392	Y = 39775X − 1881	0.9963	0.41308	0.13631	1.25 – 200
03	Vanilic Acid	15.531	Y = 65077X + 33	0.9885	0.09577	0.03192	1.25 – 250
04	Caffeic acid	16.277	Y = 72328X − 52	0.9992	0.44863	0.14804	0.5 – 320
05	Vanilin	21.46	Y = 58930X + 64	0.9933	0.11350	0.37833	4.75 – 350
06	*p*-Coumaric acid	23.817	Y = 157538X + 122	0.9996	0.09385	0.03097	4.5 – 200
07	Rutin	28.37	Y = 28144X − 24	0.9854	0.39912	0.13304	2.5 – 50
08	Naringin	34.788	Y = 19379X − 212	0.9922	0.52711	0.17570	4.25 – 80
09	Quercetin	45.047	Y = 45378X + 177	0.9988	0.13475	0.04491	2.5 – 200

LOQ: limit of quantification, LOD: limit of detection, Y: beak area, X: compound concentration.

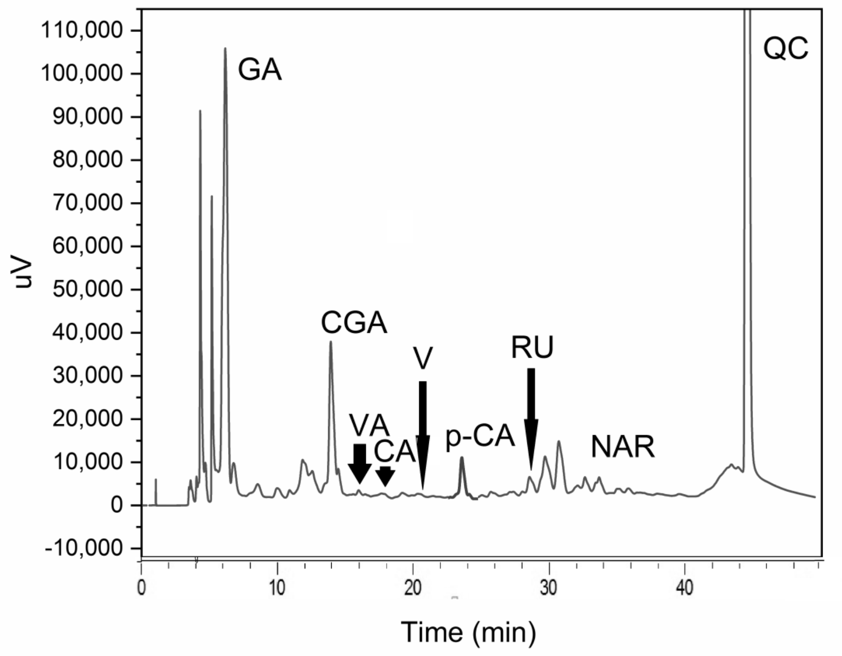

Figure S2. Phenolic compounds content identified by High-performance liquid chromatography (HPLC) chromatogram in epicarp of Djelfa —D. Identified compounds are: GA—gallic acid. CGA — Chlorogenic acid. VA—Vanillic acid. CA—Caffeic acid. V—Vanillin. p-CA—*p*-Coumaric acid. RU—Rutin. NAR—naringin and QC—quercetin.

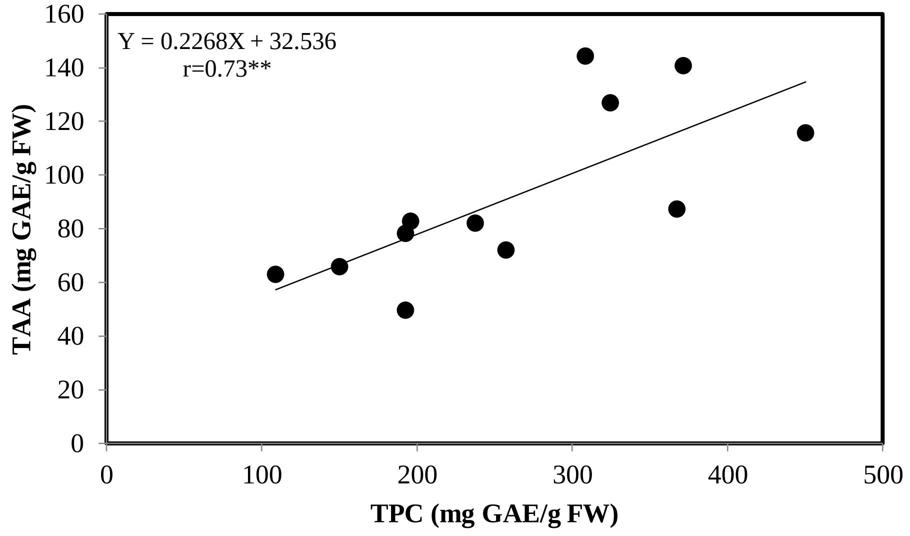

Figure S3. Correlation between antioxidant activity (TAA) and polyphenol content (TPC) in tow part of fruits of *Pistacia atlantica* Desf. harvested in six geographic areas in Algeria.

Table S2. Ecological factors of the *Pistacia atlantica* Desf. collection sites.

Site	Bioclimatic floor	Altitude (m)	Latitude (N)	Longitude (E)	Rainfall (mm)	TM (°C)	T min (°C)	T max (°C)	Soil texture	Ecology
**A**	Hyper-arid	979	32°04′6″	02°18′5″W	111	22.3	15.2	28.9	Sandy clay loam [2]	*Ziziphus lotus, Artemisia herba-alba, Retama retam*

**B**	Semi-arid	1027	35°37′10″	6°22′13″	210	16	8.3	24.1	Clay [39]	Mixedwood forest
**D**	Arid Fresh	630	34°02′11″	03°40′22″	185	15.4	9.7	21.2	Clay loam [2]	*Ziziphus lotus, Artemisia herba-alba, Stipa tenacissima*
**L**	Arid	909	34°09′11″	03°01′09″	120	20	12.8	27	-	Mixedwood forest
**T-R**	Semi-arid	808	35°22′33″	02°09′5″	190	17.7	11.4	24.9	Sandy loam [40]	*Pistacia atlantica* Desf.
**T-Z**	Semi-arid	873	34°54′45″	02°13′42″	190	17.7	11.4	24.9	-	*Ziziphus lotus, Artemisia herba-alba, Peganum harmala, marrubium vulgare*

Mean annual rainfall (mm); T M: Mean Temperature (°C); T min: Minimal Temperature (°C) and T max: Maximal Temperature (°C). Source: National Meteorology Office of Algeria (ONM) during 2016. A: Bechar, B: Batna, D: Djelfa, L: Laghouat; T-R: Tiaret-Rechaiga, T-Z: Tiaret-Zemalet El Emir Abdelkader.
